# The epidemiology of hospitalized influenza in children, a two year population-based study in the People's Republic of China

**DOI:** 10.1186/1472-6963-10-82

**Published:** 2010-03-30

**Authors:** Wei Ji, Tao Zhang, Xuelan Zhang, Lufang Jiang, Yunfang Ding, Chuangli Hao, Liwen Ju, Yuqing Wang, Qingwu Jiang, Mark Steinhoff, Steven Black, Genming Zhao

**Affiliations:** 1 Suzhou Children's Hospital, Suzhou University, Jiangsu Province, PR China; 2 Department of Epidemiology, School of Public Health, Fudan University, Key Laboratory of Public Health Safety, Ministry of Education, Shanghai, PR China; 3 Center for Global Health, Cincinnati Children's Hospital, Cincinnati, Ohio, USA

## Abstract

**Background:**

The epidemiology and disease burden of annual influenza in children in mainland People's Republic of China have not been reported in detail. To understand the incidence and epidemiology of laboratory-proven influenza hospitalization in children in China, a review of available laboratory and hospital admission data was undertaken.

**Methods:**

We conducted a retrospective population-based study in Suzhou and the surrounding area of Jiangsu province, China for hospitalized cases of respiratory illness at Suzhou Children's Hospital. Cases of pneumonia or respiratory illness were identified from hospital computer data bases. Routine virological testing by fluorescent monoclonal antibody assay of all hospitalized children identified influenza and other viruses. We calculated incidence rates using census population denominators.

**Results:**

Of 7,789 specimens obtained during 2007 and 2008, 85 were positive for influenza A and 25 for influenza B. There were 282 specimens with parainfluenza virus and 1392 with RSV. Influenza occurred throughout the year, with peaks in the winter, and in August/September. Overall estimated annual incidence of laboratory-proven influenza hospitalization was 23-27/100,000 children 0-4 years old, and 60/100,000 in infants 0-6 months, with an average hospitalization of 9 days.

**Conclusions:**

Influenza disease in young children in this part of China is a relatively common cause of hospitalization, and occurs throughout the year. The use of influenza vaccine in Chinese children has the potential to reduce the effect of influenza in the children, as well as in their communities. Studies are needed to further assess the burden of influenza, and to develop and refine effective strategies of immunization of young children in China.

## Background

Influenza is an important cause of morbidity and mortality among both children and adults. Influenza A and B viruses cause yearly epidemics with significant morbidity and mortality globally [1]. Children have the highest rates of infection while elderly adults have the highest mortality rates [2]. Influenza is also associated with substantial numbers of hospitalizations among young infants and children [3]. Neuzil et al., reported during periods when influenza virus was circulating, the rate of excess hospitalizations for cardiopulmonary conditions ranged from 104 to 9 per 10,000 children/year for children 0-59 months. Healthy infants were hospitalized for illness attributable to influenza at rates similar to those for high-risk adults [4]. A recent prospective population surveillance study in three US states confirmed these earlier findings with average annual rates of hospitalization attributable to influenza of 450 per 100,000 children/year for children less than 6 months of age (reaching annual hospitalization rates of 1% in some years). Rates/100,000 children annually ranged from 30 to 90 for those between 6-59 months of age with an overall incidence of 90. In addition, outpatient visits associated with influenza were 10 to 250 times more common than hospitalizations in younger US children [5].

For many years influenza vaccination was recommended only for children with high-risk medical conditions in the US. However, because of the high rates of hospitalization in healthy US children this recommendation was expanded in 2004-2005 to include all children 6-23 months of age and again in 2006-2007 to include all children 6-59 months of age, regardless of medical condition [6,7]. In 2008-2009, the recommendation was extended even further to include all children, aged 6 months to 18 years [8].

Several studies have shown that young children are important in the spread of community influenza since they often introduce the infection to their families, and disseminate infection through schools to the broader community [9]. In addition there is substantial evidence from the US, Italy, and Japan that immunization of young children can reduce disease rates in unimmunized members of the local community [10,11].

Most influenza strains that cause epidemic and pandemic disease appear to arise out of Asia, with East Asia being a common source [12]. Despite the importance of this region as a source of new many influenza strains, the epidemiology and burden of disease of influenza, especially in the 87 million 0-5 year old children of China has not been described. Importantly, influenza vaccination is not routinely recommended in China and immunization of children is uncommon.

**Figure 1:**
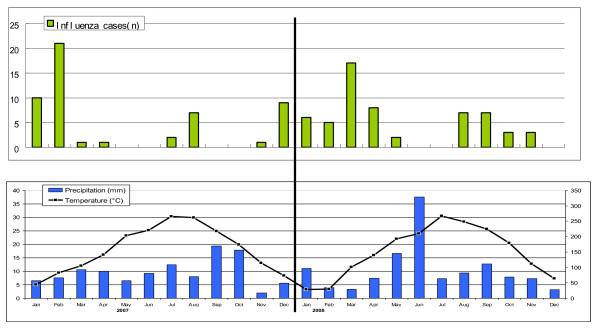
**Monthly distribution of number of influenza cases, precipitation, and mean temperature in Suzhou, China**. Mean relative humidity ranged from 60-75% over this period, and is not displayed.

In order to further the understanding of the burden and epidemiology of influenza in children in China, a review of available laboratory and hospital admission data was undertaken at the Suzhou university affiliated Children's Hospital in Jiangsu province in China. This hospital was chosen because it is the only children's hospital serving Suzhou district, which has a population of approximately 6 million people and an annual birth cohort of approximately 40,000 infants. Suzhou is located near Shanghai, approximately 31 degrees North and 120 degrees West, at a latitude similar to Jacksonville Fl in N America or Cairo, Egypt. See figure 1 for a summary of meteorological data, which is typical for a humid sub tropical latitude.

The hospital has computerized diagnostic records for all admissions, facilitating identification and characterization of all respiratory admissions and importantly has been performing virologic testing on all children admitted to the respiratory wards and ICU for the past two years.

## Methods

This was a retrospective sentinel hospital surveillance study using available clinical, laboratory and demographic data. Cases hospitalized for pneumonia were identified from computerized hospital medical records for the years 2007-2008. The Criteria for diagnosing pneumonia was a clinical diagnosis of pneumonia by the treating physician as recorded in chart. Age specific population denominators for Suzhou and the surrounding area served by the hospital were estimated from the population census for Jiangsu province for the years 2007 to 2008. Hospitalization rates were then calculated for each year for which computerized hospital data was available. For the past two years it has been the policy of the hospital to perform virologic testing routinely on all children admitted with a respiratory complaint. All children hospitalized with acute respiratory disease are admitted to one of two dedicated respiratory wards which have dedicated staff to perform this and other routine procedures. In addition, children admitted to the PICU also have routine respiratory virologic testing.

Virologic testing is performed on nasal aspirate specimens which are forwarded to the microbiology laboratory where an immuno-fluorescent kit (D^3 ^Ultra DFA Respiratory Virus Screening and Identification kit, Diagnostic Hybrids, of Athens, Ohio USA) was used according to the manufacturer's directions. This assay allows identification of adenovirus, influenza A, influenza B, parainfluenza 1, 2, and 3, and RSV viruses from clinical specimens using fluorescein-tagged monoclonal antibodies and utilizes positive and negative controls. In addition, nucleic acid from each positive specimen is banked for possible future testing.

The case count for pneumonia hospitalization was used to assess the annual epidemiology of respiratory infection in this population. In addition, the influenza data was used along with the population denominators described above to estimate the age specific rates of hospitalization for influenza in this population for the study years. These calculations assume that few local children would have been hospitalized outside of Suzhou, Jiangsu province, as this is the only children's hospital in the area.

The study was approved by the Institute Review Board in the School of Public Health at Fudan University which is registered with the office for human research protections and has a federal wide assurance (approval no. IRB #09-03-0171).

## Results

From Jan 2007 through Dec 2008, there were 7,789 specimens sent for virus screening (3,444 in 2007 and 4,345 in 2008, 63% male). All the specimens came from 0-59 month old children admitted to Suzhou Children's Hospital for respiratory infection. In total there were 95 specimens positive for influenza A and 25 for influenza B with an overall positivity rate of 1.54%, ranging up to 10 per cent in peak months. In addition, there were 282 positives for parainfluenza (types 1, 2 and 3 being 30, 2 and 250 cases respectively) and 1392 (17.87%) with RSV and 60 with adenovirus infection. It is likely that some patients 0-28 days old who were admitted to the newborn unit rather than the respiratory wards did not have routine specimens submitted for virology.

**Figure 2:**
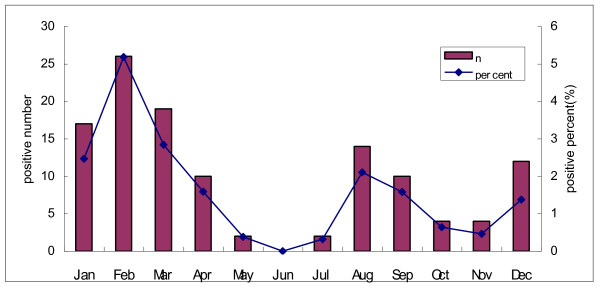
**The seasonal distribution of the influenza cases and the positive percent in 2007 and 2008 combined**.

**Figure 3:**
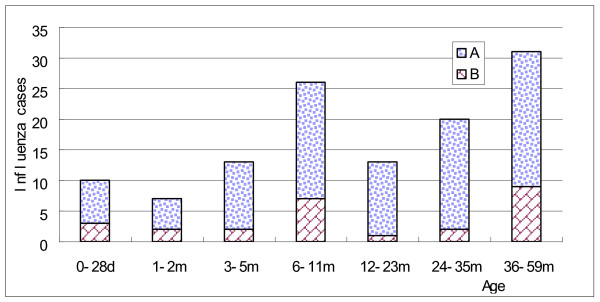
**Age distribution of confirmed influenza A and B cases, 2007 and 2008**.

**Figure 4:**
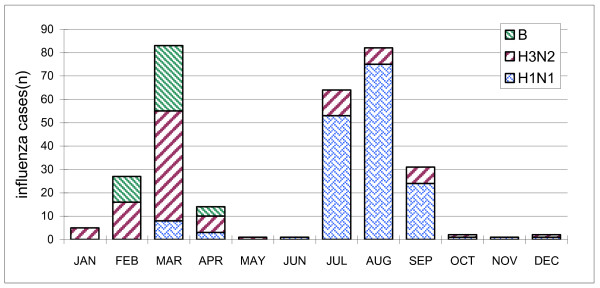
**Monthly distribution of influenza virus subtypes from nearby Shanghai in 2008**. The overall influenza seasonality is similar to Suzhou, and suggests that different subtypes may predominate in various seasons.

The seasonal distribution of the influenza cases is shown in Figure 1. Influenza cases occurred year round, for 18/24 months, most commonly in the winter and early spring (January - March) with a second late summer peak in August-September. The temperature and precipitation for the same time period in Suzhou is also shown in Figure 1. Not shown is the humidity during the same time period which was constant varying between 60-75%. The seasonal distribution of cases for both years combined is shown in Figure 2. The two peaks of disease in the winter and late summer can be more clearly seen in this figure. Hospitalization for influenza was most common in 0-6 month old infants as is shown in Figure 3 and Table 1. Figure 4 shows the monthly distribution of influenza types from nearby Shanghai for 2008, showing the distribution of influenza viruses subtypes in the winter and summer peaks which differed for this year. As shown in Table 2, the most common discharge diagnosis for hospitalized patients with proven influenza was bronchopneumonia, with pneumonia in a patient with asthma being second. Lengths of stay for children with influenza tended to be relatively long ranging between 7 and 14 days with the average being 9.42 days and 10.02 days in 2007 and 2008 respectively. Nine (8.2%) children with influenza required ICU care. All children with a confirmed diagnosis of influenza received at least one antibiotic, with beta-lactams and macrolides being the most common, respectively.

**Table 1:** Estimated age-specific annual incidence/100,000 population of all-cause pneumonia and of lab-proven influenza hospitalization

Year	Age	**Est**. **Pop**.	Total Hospitalized Children	Hospitalized Pneumonia		Influenza - Related Hospitalization	
				
				No. (death)	Incidence(95%CI)	Number	Incidence(95%CI)
2007	0-5 m	24,261	3,311	2,147(3)	8,850 (8492-9207)	15	61.8 (30.5-93.1)
	6-11 m	24,261	2,986	1,529	6,302 (5997-6608)	14	57.7 (27.5-87.9)
	12-23 m	48,522	2,302	910(2)	1,875 (1755-1996)	3	6.2 (0.8-13.2)
	24-35 m	48,522	4,556	884(2)	1,822 (1703-1941)	8	16.5 (5.1-27.9)
	36-59 m	97,044	5,014	1,311	1,351 (1278-1424)	15	15.5 (7.6-23.3)

Total	0-59 m	242,610	18,169	6,781(7)	2,795 (2729-2861)	55	22.7 (16.7-28.7)

2008	0-5 m	23,886	3,367	2,395(4)	10,027 (9646-10408)	15	62.8 (31.0-94.6)
	6-11 m	23,886	2,979	1,700(1)	7,117 (6791-7443)	12	50.2 (21.8-78.7)
	12-23 m	47,772	2,744	1,011	2,116 (1987-2245)	10	20.9 (8.0-20.9)
	24-35 m	47,772	5,433	982	2,056 (1928-2183)	12	25.1 (10.9-39.3)
	36-59 m	95,544	5,985	1,458(2)	1,526 (1448-1604)	16	16.7 (8.5-25.0)

Total	0-59 m	238,860	20,508	7,546(7)	3,159 (3089-3229)	65	27.2 (20.6-33.8)

Note: Populations estimated from the birth cohorts of 48,552 births in 2007 and 47,772 births in 2008 in Suzhou.

**Table 2:** The clinical diagnoses and selected case characteristics for the patients with confirmed influenza.

Age	N	Clinical Diagnosis					Mean Hospitalization Duration (days)	Referral ICU
		Pneumonia	Broncho-Pneumonia	Asthma with Pneumonia	Pharyngitis	Other		
0-28 d	10	10					7.5	0
1-2 m	7	1	4	1		1^A^-	10.6	2
3-5 m	13		8	5			11.2	1
6-11 m	26	3	16	5		2^B^	9.4	0
12-23 m	13	1	12				9.8	-
24-35 m	20		12	5	1	2^C^	9.0	0
36-59 m	31	2	20	3	3	3^D^	9.1	1
**Total**	**120**	**17**	**72**	**19**	**4**	**8**	**9.46**	**4**

Notes: ^A^: Broncho-pneumonia + meningitis

^B^: Bronchitis

^C^: URI

^D^: URI, meningitis, bronchitis for each

Although influenza virus sub-typing was not available for Suzhou viruses during the study period, data from Shanghai, which is 100 kilometers distant, revealed that the most common influenza subtype during the winter of 2008 was A/H3N2, during which time influenza B also circulated. In the summer of 2008, A/H1N1 predominated with no influenza B, as is shown in Figure 4.

We noted that the seasonality of influenza contrasted with the seasonality of RSV and parainfluenza. RSV occurred only in the fall and winter, whereas parainfluenza was most common in the summer months.

## Discussion

China reports 17 million births per year, about 13% of the world's birth cohort. We are not aware of a previous publication estimating the population incidence and patterns of laboratory-proven influenza in children in mainland China. A previous study estimated the rates of influenza indirectly from seasonal data of hospitalization in Hong Kong [13].

Our study data demonstrate that respiratory illness and specifically pneumonia is the most common cause of hospitalization for children under five in Suzhou, China. Overall, 2-3% of all children less than 5 years were hospitalized with a diagnosis of pneumonia. Despite the relatively high level of economic growth and development in this region, hospitalization rates are high, although mortality is very low. This would seem to indicate that the threshold for admission for respiratory illness is relatively low, as described by Chiu in Hong Kong [13]. In a planned prospective study we will evaluate the severity of illness of hospitalized children and assess the incidence of outpatient disease. Prior to the availability of this information, anecdotal information from pediatricians at the hospital would seem to confirm our impression that the one child policy in China encourages parents to seek care for their children when they are only mildly ill. However, a substantial portion of children admitted also required ICU care indicating that the case mix may be complex. Introduction of childhood influenza vaccination has the potential to reduce a substantial proportion of these influenza-associated hospitalizations.

It is of interest that influenza associated hospitalization of children occurs during 8-9 months of the year, in a perennial pattern, with two distinct annual peaks. This pattern is also reported from nearby Hangzhou, Zhejiang province [14]. This contrasts with the classical pattern of a single winter peak of influenza observed in Northern Europe and North America, and also with the year round circulation of influenza observed in tropical settings [15,16]. It is possible that this pattern may represent temporally overlapping epidemics of northern winter strains, alternating with summer strains from tropical regions and Hong Kong, as recently described by Russell et al [12]. The local virus subtype data is not available currently to evaluate this hypothesis, but the data from Shanghai shows the circulating subtypes are different each season.

The peak incidence of pneumonia hospitalization was in 0-5 month old children as reported elsewhere and in US studies. However, the annual incidence of confirmed influenza hospitalization in our study of 50-60 cases per 100,000 children < 1 year old children is substantially lower than the rates published from the US [17]. The Suzhou incidence we have calculated is substantially less than the incidence rates reported from Hong Kong. It is noted that the childhood influenza incidence data from Hong Kong derived from a seasonal analysis of excess hospitalization [18] has recently been modified by surveillance data based on influenza virus detection [13] to a fraction of former estimate.

The overall annual incidence of laboratory-proven influenza hospitalization we observed was 23-27/100,000 in 0-4 year old children. The average 10 day duration of hospitalization for influenza suggests a relatively large economic burden of childhood influenza in this region.

These rates of laboratory-proven influenza are a minimal estimate. The immunofluorescent assays we used are likely to underestimate the true number of virus infections, and a nucleic acid amplification test may provide more accurate data. The testing of patients from the respiratory wards and ICU likely missed children with influenza who were admitted to other wards with febrile syndromes.

These data show that 25% of all admitted lab-proven cases of influenza were 0-5 months old; a group whose illness can be prevented by maternal immunization [15]. Conversely, 75% were 6-59 months old, in the age group which receives influenza vaccine in the USA.

## Conclusion

Influenza disease in young children in this part of China is a relatively common cause of hospitalization, and occurs throughout the year. Since young children play a major role in the introduction and the dissemination of new influenza viruses in communities, the use of influenza vaccine in children in China has the potential to reduce the effect of influenza in the children, as well as in their communities. Studies are needed to develop and refine effective strategies of influenza immunization of young children in China.

## Competing interests

The authors declare that they have no competing interests.

## Authors' contributions

WJ participated in the design and implement of the study. TZ performed the statistical analysis and drafted the manuscript. XZ carried out the immunoassays. LJ participated in the implement of the study. YD participated in the design and implement of the study. CH participated in the design and implement of the study. LJ participated in its design and coordination. YW implemented the field study. QJ participated in the design and coordination. MS conceived the study, participated in the design of the study and helped to draft the manuscript. SB participated in its design, and coordination and drafted the manuscript. GZ conceived the idea, coordination and participated in the design. All authors read and approved the final manuscript.

## Pre-publication history

The pre-publication history for this paper can be accessed here:

http://www.biomedcentral.com/1472-6963/10/82/prepub
